# Recovering anaerobic bacterial genomes in saliva, tumour and blood whole genome sequencing data from patients with prostate cancer

**DOI:** 10.1038/s41522-026-01140-2

**Published:** 2026-10-20

**Authors:** A. Gihawi, A. Bampalis, S. Subramanian, Pan Prostate Cancer Group, Noora Al-Muftah, Esther Baena, Daniel Barrowdale, Lucy M. Barton, Rajbir N. Batra, Justin Bedő, Alain Bergeron, Alejandro Berlin, Michael Borre, Paul Boutros, G. Steven Bova, Daniel S. Brewer, Robert G. Bristow, Mark N. Brook, Benedikt Brors, Dan Burns, Adam Butler, Bethany K. Campbell, Géraldine Cancel-Tassin, Ryan Carelli, Kevin CL. Cheng, Melissa Cheung, Ken Chow, Michael J. Clarkson, Marek Cmero, Colin Collins, Colin S. Cooper, Niall M. Corcoran, Angelo Corso Faini, Tony Costello, Olivier Cussenot, Nening Dennis, Harveer Dev, Wikum Dinalankara, Ruining Dong, Philip Dundee, Ros A. Eeles, Andrew Erickson, Emre Esentürk, Fabian Falkenbach, Giuseppe N. Fanelli, Francesco Favero, Breon Feran, Angel Fernandez-Sanroman, Lars Feuerbach, Chris Foster, Yves Fradet, Michael Fraser, Yunfan Fu, Kira Furlano, Peter Georgeson, Clarissa Gerhauser, Kazzem Gheybi, Abraham Gihawi, Etsehiwot G. Girma, Vincent J. Gnanapragasam, Markus Graefen, Corinna Grima, Andreas J. Gruber, Valleriia Haberland, Grace A. Hall, Freddie Hamdy, Anis Hamid, Vanessa M. Hayes, Xiaotong He, Housheng Hansen He, Jessica Heilmann, Barbara Hernando, Vivien Holmes, Chen Hong, Matthew KH. Hong, Christopher M. Hovens, William J. Hutchison, Eddie Luidy Imada, G. Maria Jakobsdottir, Weerachai Jaratlerdsiri, Jue Jiang, Chol-hee Jung, Thomas Keane, Michael Kerger, Giselle Kerry, Francesca Khani, Daria Kiriy, Jan Korbel, Zsofia Kote-Jarai, Natalie Kurganovs, Radoslaw Lach, Alastair Lamb, Philippe Lamy, Gregory Leeman, Wing-Kit Leung, Beng Lim, Massimo Loda, Yong-Jie J. Lu, Pavlo Lutsik, Andy G. Lynch, Geoff Macintyre, Stefano Mangiola, Luigi Marchionni, Charlie E. Massie, Patrick McCoy, Arfa Mehmood, Nana Mensah, Ian Mills, Toby Milne-Clark, Ramyar Molania, Anne Nguyen, Sam Norden, Pier Vitale Nuzzo, Matti Nykter, Samson Olofinsae, Andre V. Olsen, Mohamed Omar, Simon Pacey, Hubert Pakula, Yidan Pan, Anthony T. Papenfuss, Daniel J. Park, Filippo Pederzoli, Diogo Pellegrina, Jocelyn Penington, Justin S. Peters, Parmit M. Phal, Parsa Pirhady, Christoph Plass, Bernard Pope, Lucio R. Queiroz, Keiran Raine, Tobias Rausch, Jüri Reimand, Brian Robinson, Silvia Rodrigues, Ronnie Rodrigues Pereira, Alan F. Rubin, Andrew Ryan, Atef Sahli, Chris Sander, Aravind Sankar, Guido Sauter, Michael Scherer, Thorsten Schlomm, Balthasar C. Schlotmann, Ronald Simon, Pamela X. Y. Soh, Karina D. Sørensen, Ryan Stuchbery, Holger Sültmann, Avigail Taylor, Pierre Tennstedt, Gabriel Tulcan, Sebastian Uhrig, Benedicte P. Ulhoi, Jennifer Ureta, Theo Van Der Kwast, Peter Van Loo, Clare Verrill, Tapio Visakorpi, Lena Voith von Voithenberg, Matthew Wakefield, Anne Warren, David C. Wedge, Joachim Weischenfeldt, Christopher Wirth, Dan J. Woodcock, Yaobo Xu, Takafumi N. Yamaguchi, Claudio Zanettini, Jingjing Zhang, Aleksandra S. Ziuboniewicz, G. S. Bova, A. Butler, N. M. Corcoran, F. Favero, V. Gnanapragasam, A. Hamid, C. Jung, Z. Kote Jarai, G. Leeman, M. Loda, J. Reimand, A. Sahli, T. Schlomm, T. N. Yamaguchi, J. Weischenfelft, C. Hovens, B. Pope, R. Bristow, M. N. Brook, K. C. L. Cheng, E. G. Girma, A. J. Gruber, H. H. He, E. L. Imada, G. M. Jakobsdottir, F. Khani, P. Lutsik, L. Marchionni, R. Molania, A. T. Papenfuss, D. Pellegrina, L. R. Queiroz, T. Rausch, B. Robinson, C. Zanettini, K. D. Sørensen, P. Lamy, G. Cancel-Tassin, O. Cussenot, R. A. Eeles, N. Annels, V. M. Hayes, R. Hurst, C. S. Cooper, D. S. Brewer

**Affiliations:** 1 Department of Metabolic Health, Norwich Medical School, University of East Anglia, University of East Anglia, Norwich, UK; 2 Ancestry and Health Genomics Laboratory, Charles Perkins Centre, School of Medical Sciences, Faculty of Medicine and Health, University of Sydney, Camperdown, NSW Australia; 3 Prostate Cancer Research Center, Faculty of Medicine and Health Technology, Tampere University, Tampere, Finland; 4 Wellcome Sanger Institute, Cambridge, UK; 5 Department of Surgery, The University of Melbourne, Parkville, VIC Australia; 6 Department of Urology, Peninsula Health, Frankston, VIC Australia; 7 Division of Urology, Royal Melbourne Hospital, Parkville, VIC Australia; 8 The Walter and Eliza Hall Institute of Medical Research, Parkville, VIC Australia; 9 Department of Urology, Western Health, Footscray, VIC Australia; 10 Biotech Research & Innovation Centre (BRIC), University of Copenhagen, Copenhagen, Denmark; 11 Finsen Laboratory, Copenhagen University Hospital—Rigshospitalet, Copenhagen, Denmark; 12 Cambridge Prostate Cancer Research and Clinical Trials Group, University of Cambridge & Cambridge University Hospitals NHS Trust S2, Cambridge Biomedical Campus, Cambridge, UK; 13 Genitourinary Oncology Service, Memorial Sloan Kettering Cancer Center, New York, NY USA; 14 Melbourne Bioinformatics, The University of Melbourne, Parkville, VIC Australia; 15 Royal Marsden NHS Foundation Trust, London, UK; 16 The Institute of Cancer Research, London, UK; 17 Department of Pathology and Laboratory Medicine, Weill Cornell Medicine, New York, NY USA; 18 The Broad Institute of Harvard and MIT, Cambridge, MA USA; 19 The New York Genome Center, New York, NY USA; 20 Computational Biology Program, Ontario Institute for Cancer Research (OICR), Toronto, ON Canada; 21 Department of Medical Biophysics, University of Toronto, Toronto, ON Canada; 22 Department of Molecular Genetics, University of Toronto, Toronto, ON Canada; 23 Manchester Cancer Research Centre, The University of Manchester, Manchester, UK; 24 Department of Urology, Charité—Universitätsmedizin Berlin, Corporate Member of Freie Universität Berlin and Humboldt Universität zu Berlin, Berlin, Germany; 25 Jonsson Comprehensive Cancer Centere, University of California—Los Angeles, Los Angeles, CA USA; 26 Collaborative Centre for Genomic Cancer Medicine, The Victorian Comprehensive Cancer Centre, Parkville, VIC Australia; 27 Department of Clinical Pathology, The University of Melbourne, Parkville, VIC Australia; 28 Australian BioCommons, The University of Melbourne, North Melbourne, VIC Australia; 29 Faculty of Medicine Nursing and Health Sciences, Department of Medicine, Central Clinical School, Monash University, Melbourne, VIC Australia; 30 Christie NHS Foundation Trust, Manchester, UK; 31 Div Cancer Sciences, FBMH, University of Manchester, Manchester, UK; 32 Manchester Cancer Research Centre and Cancer Research UK Manchester Institute, The University of Manchester, Manchester, UK; 33 Department of Biology, University of Konstanz, Konstanz, Germany; 34 Princess Margaret Cancer Centre, University Health Network, Department of Medical Biophysics, University of Toronto, Toronto, ON Canada; 35 Division of Cancer Sciences, The University of Manchester, Manchester, UK; 36 Division of Cancer Epigenomics, German Cancer Research Center (DKFZ), Heidelberg, Germany; 37 Department of Oncology, KU Leuven, Leuven, Belgium; 38 Department of Medical Biology, The University of Melbourne, Melbourne, VIC Australia; 39 Genome Biology Unit, European Molecular Biology Laboratory (EMBL), Heidelberg, Germany; 40 Department of Clinical Medicine, Aarhus University & Department of Molecular Medicine, Aarhus University Hospital, Aarhus, Denmark; 41 CeRePP (Centre de Recherche sur les Pathologies Prostatiques et Urologiques), Paris, France; 42 Sorbonne Université, GRC n°5 Predictive Onco-Urology, APHP, Tenon Hospital, Paris, France; 43 University of Surrey, Surrey, UK; 44 School of Health Systems and Public Health, University of Pretoria, Pretoria, South Africa; 45 The Earlham Institute, Norwich, UK; 46 Nuffield Department of Surgical Sciences, John Radcliffe Hospital, Oxford, UK; 47 European Bioinformatics Institute (EMBL-EBI), Cambridge, UK; 48 Cancer Research UK Manchester Institute, The University of Manchester, Manchester, UK; 49 Division of Cancer Sciences, FBMH, SMS, University of Manchester, Manchester, UK; 50 Department of Oncology, Early Cancer Institute, University of Cambridge, Cambridge, UK; 51 German Cancer Research Center (DKFZ, Heidelberg, Germany; 52 Research Center, Oncology Axis, CHU de Québec—Université Laval, Québec, QC Canada; 53 Cancer Research Center, Laval University, Québec, QC Canada; 54 Department of Surgery, Laval University, Québec, QC Canada; 55 University Health Network, Toronto, ON Canada; 56 Department of Urology, Aarhus University Hospital, Aarhus, Denmark; 57 Department of Clinical Medicine, Aarhus University, Aarhus, Denmark; 58 The Earlham Institute, Norwich Research Park, Norwich, UK; 59 Division Cancer Sciences, Faculty of Biology, Medicine & Health, University of Manchester, Manchester, UK; 60 Division Applied Bioinformatics, German Cancer Research Center (DKFZ), Heidelberg, Germany; 61 German Cancer Consortium (DKTK), Core Center Heidelberg and National Center for Tumor Diseases (NCT), Heidelberg, Germany; 62 Medical Faculty Heidelberg and Faculty of Biosciences, Heidelberg University, Heidelberg, Germany; 63 Department of Surgery, The Collaborative Centre for Genomic Cancer Medicine, The University of Melbourne, Parkville, VIC Australia; 64 Weill Cornell Medical College, New York, NY USA; 65 Computational Biology Program, Ontario Institute for Cancer Research, Toronto, ON Canada; 66 Department of Oncology, Early Cancer Institute, University of Cambridge, Box 197, Cambridge Biomedical Campus, Cambridge CB2 0XZ, Box 197, Cambridge Biomedical Campus, Cambridge, UK; 67 Vancouver Prostate Centre, Vancouver, BC Canada; 68 Department of Urology, Royal Melbourne Hospital, Parkville, VIC Australia; 69 Australian Prostate Cancer Centre, Melbourne, VIC Australia; 70 Department of Pathology and Laboratory Medicine, Weill Cornell Medical College, Weill Cornell Medical College, New York, NY USA; 71 School of Computing and Information Systems, The University of Melbourne, Parkville, VIC Australia; 72 The Australian Medical Robotics Academy, North Melbourne, VIC Australia; 73 The Royal Marsden NHS Foundation Trust, London & Sutton, UK; 74 Nuffield Department of Surgical Sciences, University of Oxford, Oxford, UK; 75 Prostate Cancer Center, Martini-Klinik am Universitätsklinikum Hamburg-Eppendorf, Hamburg, Germany; 76 Pathology and Laboratory Medicine, Weill Cornell Medicine (and University of Pisa, Pisa, Italy), New York, NY USA; 77 Spanish National Cancer Research Centre (CNIO), C/Melchor Fernández Almagro, Madrid, Spain; 78 Princess Margaret Cancer Centre, University Health Network, University of Toronto, Toronto, ON Canada; 79 Department of Urology, Charité—Universitätsmedizin Berlin, Berlin, Germany; 80 Clinical Pathology, The University of Melbourne, Parkville, VIC Australia; 81 The University of Melbourne Centre for Cancer Research, Victorian Comprehensive Cancer Centre, Melbourne, VIC Australia; 82 Division Cancer Epigenomics, German Cancer Research Center (DKFZ), Heidelberg, Germany; 83 School of Medical Sciences, University of Sydney, Faculty of Medicine & Health, Level 3, Charles Perkins Centre D17, Camperdown, NSW Australia; 84 Department of Surgery, Urology, University of Cambridge & Cambridge University Hospitals NHS Trust, Cambridge Urology Translational Research and Clinical Trials (Office), Cambridge Biomedical Campus, Addenbrooke’s Hospital Site, Cambridge, UK; 85 University of Konstanz, Konstanz, Germany; 86 School of Health Systems & Public Health, University of Pretoria, Pretoria, South Africa; 87 Manchester Cancer Research Centre, University of Manchester, Manchester, UK; 88 Division Molecular Genetics, German Cancer Research Center (DKFZ), Heidelberg, Germany; 89 Christie Hospital, The Christie NHS Foundation Trust, Manchester Academic Health Science Centre, Manchester, UK; 90 Genome Biology, European Molecular Biology Laboratory (EMBL), Heidelberg, Germany; 91 Barts Cancer Institute, Queen Mary University of London, Oxford, UK; 92 Department of Molecular Medicine, Aarhus University Hospital, Aarhus, Denmark; 93 CRUK Cambridge Institute, Hutchison MRC Research Centre, University of Cambridge, Li Ka Shing Centre, Cambridge Biomedical Campus, Cambridge, UK; 94 Department of Radiology, The University of Melbourne, Parkville, VIC Australia; 95 Barts Cancer Institute, Queen Mary University of London, London, UK; 96 School of Medicine, School of Mathematics & Statistics, University of St Andrews, Fife, UK; 97 Early Cancer Institute, Department of Oncology, University of Cambridge, Box 197, Cambridge Biomedical Campus, Cambridge, UK; 98 The Francis Crick Institution, The Francis Crick Institution, London, UK; 99 TissuPath, Mt Waverly, VIC Australia; 100 Pathology and Laboratory Medicine, Weill Cornell Medical College, New York, NY USA; 101 The University of Texas M.D. Anderson Cancer Center, Houston, TX USA; 102 Melbourne Bioinformatics, The University of Melbourne, North Melbourne, VIC Australia; 103 Department of Biochemistry & Pharmacology, The University of Melbourne, Melbourne, VIC Australia; 104 Australian Prostate Cancer Centre, North Melbourne, VIC Australia; 105 Manchester Cancer Research Centre and CRUK Manchester Institute, The University of Manchester, Manchester, UK; 106 Genome Biology, European Molecular Biology Laboratory (EMBL), Heidelberg, Germany; 107 cBio Center, Dana-Farber Cancer Institute, Boston, MA USA; 108 Prostate Cancer Center, Martini-Klinik, University Medical Centre Hamburg—Eppendorf (UKE), Hamburg, Germany; 109 Division Cancer Genome Research, German Cancer Research Center (DKFZ), Heidelberg, Germany; 110 National Center for Tumor Diseases (NCT), Heidelberg, Germany; 111 Department of Pathology, Aarhus University Hospital, Aarhus, Denmark; 112 The University of Texas, M.D. Anderson Cancer Center, Houston, TX USA; 113 The Francis Crick Institution, London, UK; 114 Institute of Biosciences and Medical Technology, BioMediTech, University of Tampere & Fimlab Laboratories, Tampere, Finland; 115 Cambridge University Hospitals NHS FT & University of Cambridge, Cambridge Biomedical Campus, Cambridge, UK

**Keywords:** Metagenomics, Microbial communities, Microbial genetics

## Abstract

Microbial constituents in the gastrointestinal tract, the urine and prostate tissue are being linked with clinical differences in prostate cancer, and a recent meta-analysis has linked periodontal disease to this cancer. Several hypotheses exist regarding how specific bacteria can contribute towards the development of prostate cancer. Recovering microbial genomes, and their genomic features, from clinical samples and investigating fine-scale taxonomy is a critical first step in investigating causality. Here, we describe the microbial diversity of samples associated with prostate cancer. Metagenomic assembly of non-human reads from the PanProstate Cancer Group, derived from blood, saliva samples and prostate tissue whole-genome data (*N* = 247), successfully reconstructed 249 highly complete bacterial genomes, predominantly from oral samples. Our data, reveals eighteen bacterial species from the oral cavity without species level classification, while demonstrating a wide range of known species. This approach enables us to gain additional insights of the microbes in cancer patients at no additional sequencing cost and has the potential to reveal new links that could improve our understanding of cancer initiation and development.

## Introduction

Prostate cancer was the second most frequent cancer and the fifth highest cause of cancer mortality in 2022, with an estimated 1.5 million new cases and 397,000 deaths in 2022^1^. Recognised as a hallmark of cancer^2^, the microbiome and dysbiosis is emerging as a contributing factor in prostate cancer^3,4^. Previously, we reported on bacteria detected in prostate tissue and urine^5^. Identifying the presence of at least one genus from an anaerobic bacterial biomarker set (ABBS) discerned a subset of men with a higher risk of PSA progression, metastasis or death^5^. Several unknown species were also identified by culture and sequencing^5^, suggesting that many more remain to be discovered when considering larger sample sizes and a wider geographical reach.

Although a relationship between bacteria and aggressive prostate cancer has been identified, it is not yet clear if this association is underpinned by a causal relationship. Several lines of evidence exist to suggest that this may be the case. The bacterial genotoxin colibactin, associated with DNA damage and *TMPRSS2-ERG* fusions may contribute to a subset of cancers^6^. It is also unclear how microbial genotoxic products might interact with germline DNA repair insufficiency^7,8^. Other potentially causal mechanisms of bacterial mediated tumorigenesis have been proposed, stretching across the hallmarks of cancer, including activation of host cell signalling, immunomodulation, altered metabolism and more^9^. A comprehensive description of the microbial diversity found in prostate cancer samples is required to facilitate investigations into causality.

There is also accumulating evidence linking periodontal disease to the development of human cancer. Evidence was initially found for pancreatic, head and neck and lung cancer^10–12^, but more recently for prostate cancer^13,14^. These results are significant because a major aetiological factor in the development of periodontal disease is the build-up of bacteria-containing plaque^15^. Bacteria present in the gastrointestinal tract are also linked to prostate cancer development. Faecal microbiota transplantation (FMT) from castration-resistant prostate cancer mice was sufficient to induce castration resistance consistent with the observation that gut bacteria can produce testosterone^13^. Antibiotics or FMT from hormone-sensitive patients also restricted tumour growth, suggesting a potentially clinically useful approach^13^. Additional evidence for this link is provided by numerous studies investigating the potential functional effects of the gastro-intestinal microbiome and its constituent microorganisms^9,16–19^.

Following flagship projects such as the International Cancer Genome Consortium (ICGC)^20^ and The Cancer Genome Atlas (TCGA)^21^ the availability of sequencing data from human cancer patients alongside the availability of microbial reference genomes and of taxonomic classification software has resulted in the widespread study of microbes and their complex associations with malignancy. The comprehensive characterisation of bacteria using sequencing data from samples intended for sequencing human DNA (tissue or germline samples) comes with a distinct set of technical challenges when compared with more traditional microbiome research of the gut/stool. Firstly, human tissue sequencing projects are not typically designed for microbial analysis meaning that sequencing protocols are not optimised for microbial recovery, and have limited access to population-matched control samples. Secondly, such datasets are also dominated by human DNA sequences, although this can be diminished computationally by identifying sequencing reads with constituent human *k*-mers or by removing reads that align to the human genome. Previous investigations of microbes from human ‘omics data have used reference-dependent approaches to look for clade specific *k*-mers in tumour sequencing data and have reported at the genus level^22^. Typically, these approaches do not perform well at species level^23^, can hold misclassified sequencing reads^24,25^, pay different amounts of attention to the contribution of contamination^26,27^ and are limited in depicting the fine-scale genomic differences between bacterial genomes associated with disease.

Metagenomic assembly, on the other hand, can have fewer false positive classifications but is limited by the requirement to generate a complete assembly, which is particularly challenging for low-abundance microbes or samples with multiple related strains^28^. Recent software developments have permitted the quality control and assessment of metagenome-assembled genomes (MAGs) from biological samples, most notably CheckM2^29,30^ and GUNC^31^. These tools have facilitated the discovery of microorganisms and have proven beneficial in unveiling those that are particularly difficult to isolate from clinical samples^32–36^.

Because of these developments, numerous studies, using a variety of computational tools have emerged detailing the in-silico recovery of bacterial genomes^37–39^ which has greatly expanded our knowledge of the tree of life. Typically, these studies benefit from sequencing protocols optimised for microbial DNA recovery. We set out to determine whether it was possible to identify complete bacterial genomes hiding in ‘plain sight’ (with no additional cost) by applying metagenomic assembly to the unmapped sequencing reads from whole genome sequenced data from tumour, blood and salivary samples taken from cancer patients using prostate cancer as an example. By doing so, we contribute to the known pool of microbial genomes recovered from patients with prostate cancer, permitting future investigations into microbial genes that may be causally related.

## Results

### Metagenomic assembly on non-human reads from human WGS data reveals putative microbial genomes with estimated high completeness and low contamination

The Pan Prostate Cancer Group (PPCG) Consortium has assembled a clinically well-annotated and diverse collection of whole-genome sequencing (WGS) data from fresh frozen cancers and corresponding germline samples taken from 1001 patients. The sequencing data has been reanalysed with standardised, high-accuracy pipelines annotating high-accuracy somatic variant calls, driver mutations and mutational signatures^40^.

The taxonomic classification of sequencing reads unmapped to the human genome was investigated from within *N* = 2176 WGS datasets from tumour, morphologically normal and blood/saliva germline samples. Samples with evidence for at least one of the genera: *Ezakiella*, *Fenollaria*, *Peptoniphilus*, *Porphyromonas*, *Anaerococcus* and *Fusobacterium* were selected for metagenomic assembly (Supplementary Fig. S1 provides a workflow summary diagram). These genera were selected because they comprise the ABBS and have previously been associated with the development of aggressive prostate cancer^5^. This yielded WGS data from 247 samples and 194 patients. These samples include 165 primary tumour samples, 62 germline samples and 13 metastatic tumours (with 7 unknown types). Tissue types in this sample set include 165 prostate samples, 24 saliva, 8 lymph node, 8 blood, 3 bone, 1 lymph node and 1 soft tissue (37 unknown).

The quality-trimmed unmapped reads from each of these samples were subject to metagenomic assembly (MetaSPAdes^37^). The median number of reads per sample was 4,952,049 (Q1:Q3 2,949,908, 9,987,724). There was limited difference between primary (median: 4,751,178, Q1–Q3: 3,153,538–7,877,079) and metastatic tumours (median: 11,193,600, Q1–Q3: 4,240,583–12,354,076) (Wilcoxon rank sum test *P* = 0.13). Saliva samples however, contained statistically significantly more unmapped reads (Median: 59,184,701, Q1–Q3: 35,283,741–107,635,982) than primary tumours, consistent with analysis seen in other studies (Wilcoxon rank-sum test *P* = 4.58 × 10^−15^)^41^.

**Fig. 1:**
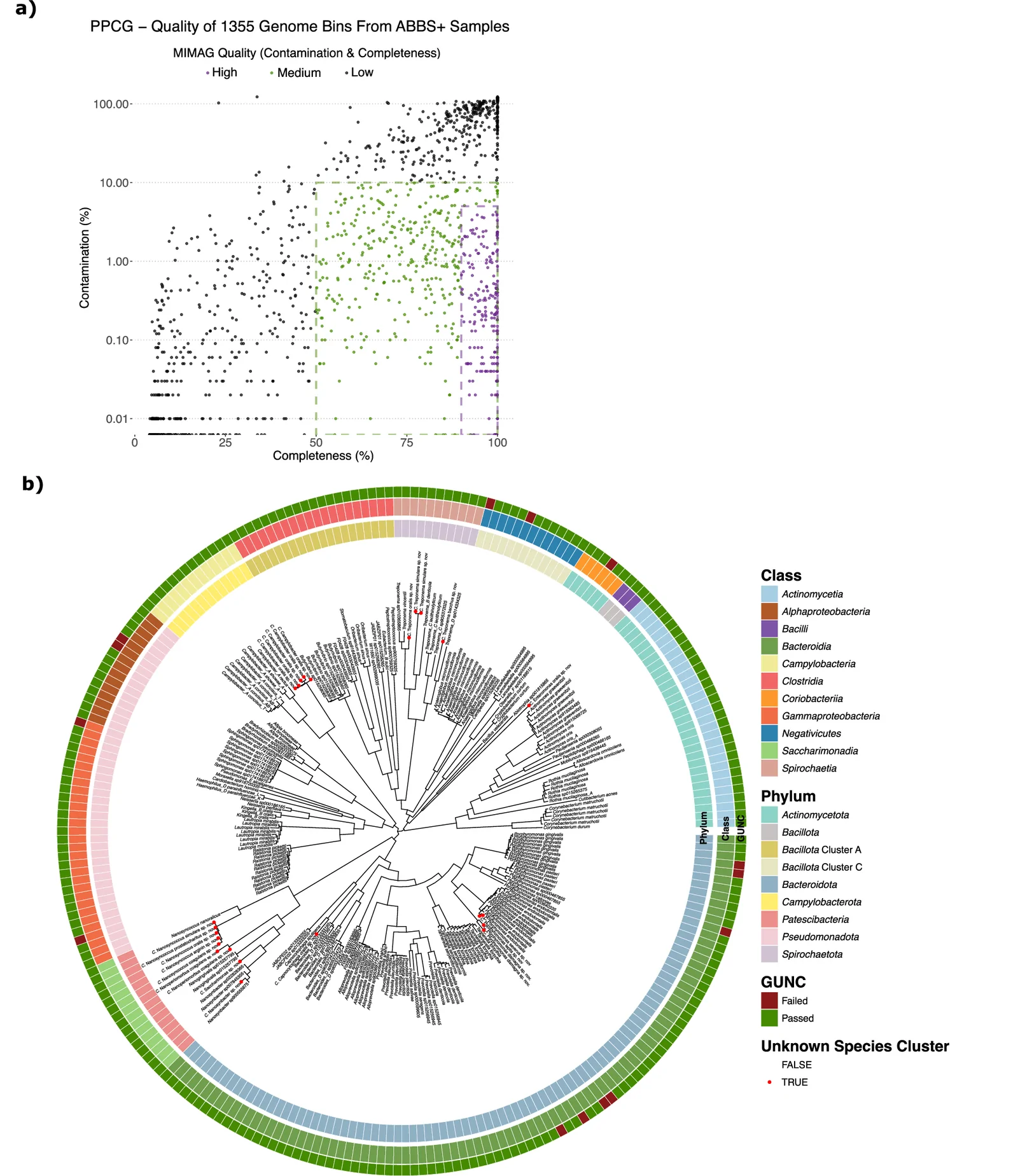
Bins benerated from samples positive for members of the Anaerobic Bacterial Biomarker Set (ABBS). **a** MIMAG standards (in terms of completeness and contamination) estimated by CheckM for 1355 putative genome bins from a subset of 247 samples from the Pan-Prostate Cancer Group (PPCG). Estimates of completeness and contamination are shown on the x and y axes respectively. High-quality bins are shown in purple (*n* = 212, >90% completeness, <5% contamination, excluding tRNA/rRNA considerations). 349 Medium-quality bins are shown in green (≥50% completeness, <10% contamination). 794 low-quality genome bins are shown in black (completeness <50%, contamination >10%). See Supplementary Data 1 for full results. **b** A phylogenetic tree of 249 MAGs that were deemed to have high-quality characteristics with CheckM (Version 1 or 2). Genomes without species cluster representatives are demonstrated with red circles at the tips of the tree. Phylum, class and the status of the GUNC test are shown on the circumference of the tree. The tree was generated with PhyloPhlAn (version 3.1.68) and IQTREE3 (3.1.1) before plotting with ggtree.

10.2 million contigs from all samples were produced. Contigs from each sample were binned (MetaBAT) to form 1355 putative genome bins. Completeness and contamination were estimated with CheckM (version 1 and 2)^29,30^ and sorted into low, medium and high-quality like bins based on MIMAG standards of contamination and completeness^38^ (Fig. 1, Supplementary Data 1). 249 genomes in total were identified as having >90% completeness and <5% contamination (212 by CheckM version 2, Fig. 1).

### Known genomes isolated from high-quality like genome bins

We assigned taxonomy to the 249 putative genome bins with high completeness/low contamination using whole genome composition (using GTDB-Tk^39^; Supplementary Data 2 and 3). *Porphyromonas* was the most frequently recovered genus (35 genomes). The next most frequently recovered genera were *Prevotella* (21 genomes), *Tannerella* (13 genomes), *Ralstonia* (12 genomes), *Actinomyces* (11 genomes) and *Anaeroglobus* (11 genomes). Most good quality MAGs (high completeness/low contamination) originated from saliva samples from France (86%). 21 were obtained from tumour samples, from the genera: *Ralstonia* (8), *Sphingomonas* (8), *Afipia* (2), *Bradyrhizobium* (2), *Bacillus* (1).

### Porphyromonas metagenome assembled genomes (MAGs)

*Porphyromonas* is an important Gram-negative anaerobic genus in the human microbiome that contains pathogenic species. It is typically known as an oral pathobiont^15^ but has been associated with progression in various diseases, including prostate cancer^3,42^. It has also been simultaneously detected in subgingval plaque and prostatic fluid in patients with concurrent periodontitis and benign prostatic hyperplasia, which it has been suggested to exacerbate^43^. New species have been discovered recently, suggesting that the full diversity of the *Porphyromonas* genus has not yet been fully elucidated^5^. In this study, it was the most frequently recovered highly complete MAG with 35 genomes recovered (Table 1). Of the 35 genomes recovered, 12 were assigned to the species *P. endodontalis*, 10 to *P. gingivalis*, 5 to *P. pasteri*, 2 to *Porphyromonas sp000467855*, 1 to *P. catoniae* and 1 to *Porphyromonas sp003640335*. For four MAGs, no taxonomic assignment was made (Table 2).

**Table 1:** Taxonomic assignments at genus level using GTDB-Tk

Genus (Based on GTDB Taxonomy)	Number of Genomes	Sample Type, Malignant Prostate tumour or blood/saliva sample collected for germline sequencing (*n*)	Originating Cohort (*n*)	Identified in Negative Controls (Salter et al.)^26^
*Porphyromonas*	35	Saliva (34)	France (34)	
*Prevotella*	21	Saliva (20)	France (20)	
*Tannerella*	13	Saliva (13)	France (13)	
*Ralstonia*	12	Blood (4) Tumour (8)	Australia (1) Canada (11)	Yes
*Actinomyces*	11	Saliva (11)	France (11)	
*Anaeroglobus*	11	Saliva (11)	France (11)	
*Cluster D within the Bacteroides genus*	9	Saliva (9)	France (9)	
*Sphingomonas*	9	Tumour (8)	Australia (1) Canada (7)	Yes
*Alloprevotella*	8	Saliva (8)	France (8)	
*Cluster A within the Campylobacter genus*	8	Saliva (8)	France (8)	
*Lautropia*	8	Saliva (8)	France (8)	
*Butyrivibrio*	7	Saliva (7)	France (7)	
*Corynebacterium*	6	Saliva (6)	France (6)	Yes
*Nanosynococcus*	6	Saliva (6)	France (6)	
*Rothia*	6	Saliva (6)	France (6)	
*Cluster B within the Campylobacter genus*	5	Saliva (5)	France (5)	

Bacterial genera previously reported in negative controls as in Salter et al.^26^ are highlighted.

Genera with less than five assembled genomes have been omitted for clarity. These genera are: *Afipia*, *Bradyrhizobium*, cluster F0058 within the *Paludibacteraceae* family, cluster F0428 within the *Lachnospiraceae* family, cluster B within the *Kingella*genus, *Lancefieldella*, *Treponema*, cluster C within the *Treponema* genus, *Alloscardovia*, *Capnocytophaga*, *Cryptobacterium*, Cluster GCA-2749205 within the *Treponemaceae* family, cluster D within the *Haemophilus* genus, cluster JABCPE02 within the *Weeksellaceae* family, Oribacterium, Pauljensenia, Peptostreptococcus, Cluster D within the Treponema genus, Abiotrophia, Bacillus, Cardiobacterium, Centipeda, Cutibacterium, Enteromonas, Cluster B within the Eubacterium_B genus, *Mobiluncus*, *Moraxella*, *Neisseria*, Cluster F within the *Olsenella* genus, *Peptidiphaga*, Cluster E within the *Pseudomonas* genus, *Stomatobaculum*, cluster B within the *Treponema* genus and *Veillonella*. Germline samples were from saliva (France) or from whole blood otherwise. See Supplementary Data 3 for the full table.

**Table 2:** The number of putative unknown species identified from 249 metagenome assembled genomes (MAGs) from the Pan-Prostate Cancer Group with high completeness and low contamination

Genus (GTDB Taxonomy)	Number of Putative *Candidatus* Unknown Genomes	Number of Unique Species
Cluster B within the *Campylobacter* genus	5	1
*Nanosyncoccus*	5	5
*Porphyromonas*	4	3
Cluster GCA-2749205 within the *Treponema* genus/*Treponemaceae* family	2	1
*Nanoperiomorbus*	2	2
*Capnocytophaga*	1	1
*Enteromonas*	1	1
*Nanosynbacter*	1	1
*Saccharimonas*	1	1
*Treponema*	1	1
Cluster D within the *Treponema* genus	1	1
Total	24	18

The number of unique species within each genus was determined using orthologous average nucleotide identity^67^ > 95% (default GTDB-Tk settings). All unknown genomes were isolated from saliva samples.

We sought to investigate how well the *Porphyromonas* MAGs from this study represent their matched reference genomes. The *P. endodontalis* MAGs contained high average nucleotide identities (as measured with fastANI within GTDB-Tk) ranging from 99.93% to 95.31% (mean 96.76%, Q1–Q3: 96.33%–96.75%). One *P. endodontalis* MAG (EGAF00001796793.17) demonstrating a particularly high ANI (99.93%) was compared to the *Porphyromonas endodontalis* reference genome (GCF_000174815.1) using progressiveMauve^44^ (Supplementary Fig. S2). Only four gaps of a total 18,747 bp were reported missing in *Porphyromonas* MAGs compared to the reference genome (Supplementary Fig. S1). This represents 0.9% of the 2,064,508 bp reference genome and included 54/1677 gene annotations, 20 of which were estimated to be essential in related species *Porphyromonas gingivalis*^45^. All of these essential genes apart from one (*dnaX*) were annotated elsewhere in the MAG. This suggests that the missing regions from the reference strain may be due to natural and/or structural variation and showing that the reference sequence is well represented by the MAG.

### Unidentified candidatus bacterial species

Unidentified Candidatus species were considered to be found when GTDB-Tk failed to identify an existing organism with an average nucleotide identity of 95% or greater (default parameters). Overall 24/249 (9.6%) recovered highly complete microbial genomes met this criteria, were not classified in existing species clusters and were considered as unique, all originating from saliva samples^39^. These putative unknown species had estimated completeness with a median of 94.47% (Q1–Q3: 92.27–99.23), and a median contamination estimate of 0.24% (Q1–Q3: 0.03–0.69). The most commonly recovered unknown candidatus species belongs to the *Campylobacter_B* genus representing a single species recovered from five different patients (Supplementary Fig. S3). Additionally, five genomes belonging to *Nanosyncoccus* were recovered.

**Fig. 2:**
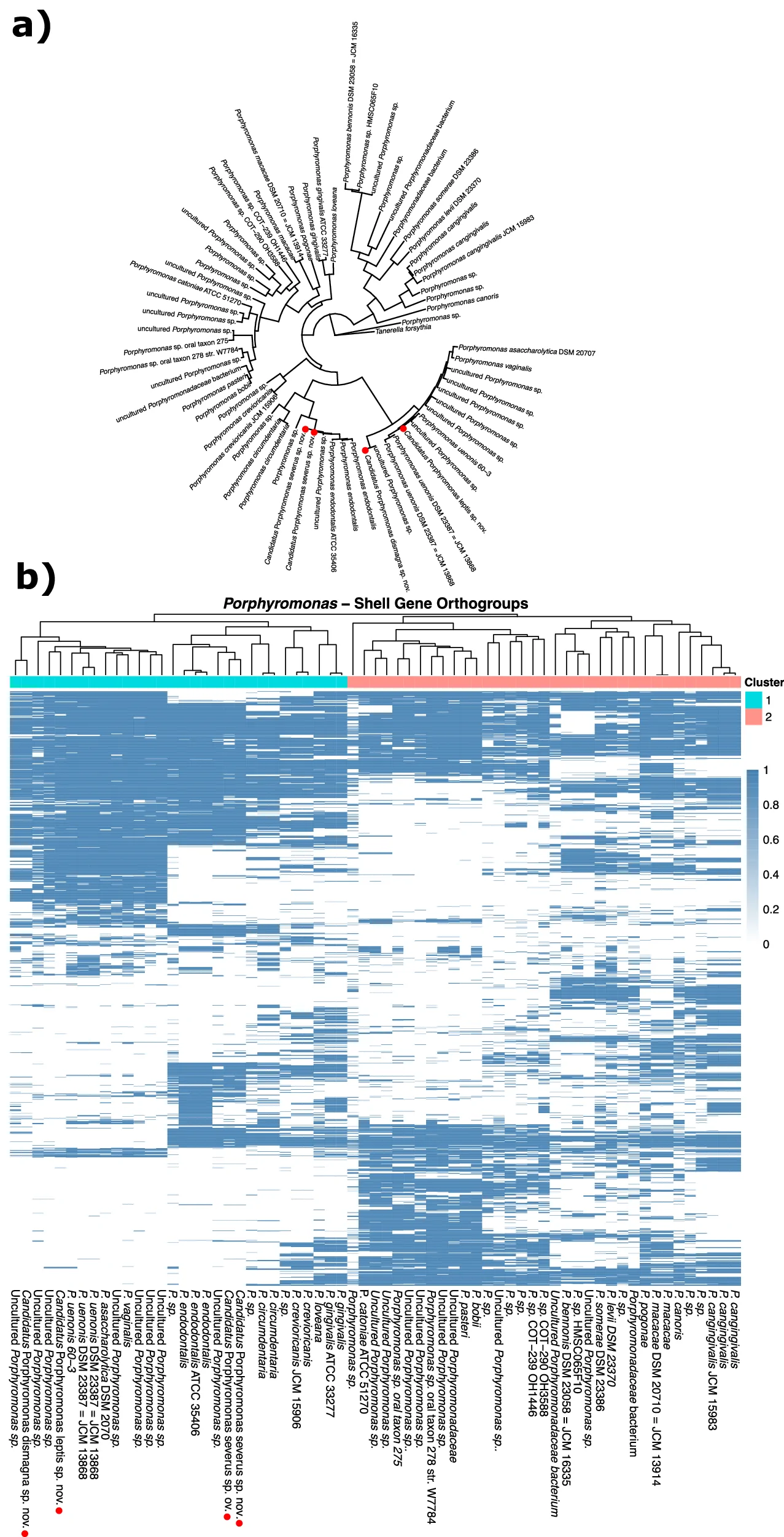
Investigating assembled *Porphyromonas* species. **a** A phylogenetic tree of *Porphyromonas* species representatives (GTDB) and species isolated in this study rooted using Tanerella forsythia. The tree was generated by first checking completeness with CheckM2, aligning marker genes with PhyloPhlAn (version 3.1.68), generating a tree file with Iqtree3 (version 3.1.1) and plotting with the ggtree and ggtreeExtra R packages. **b** Presence of shell orthogroups in *Porphyromonas* species representatives. Orthogroups and genomes were subject to hierarchical clustering and the genomes divided into two clusters (blue and red). Taxonomy is as according to NCBI. The exact genomes used are displayed in Supplementary Fig. S8.

Generally, the *Porphyromonas* genus appears diverse with a median average nucleotide identity between orthologues of 66.24%. Four distinct putative unknown *Porphyromonas* genomes were detected. Two highly similar MAGs (Sample IDs: EGAF00001796737.18 & EGAF00001796765.16) represented a single species (ANI = 97.82%) and exhibited 91% and 90% orthologous average nucleotide identity, respectively, with *P. endodontalis* as the most closely related known species. The remaining two genomes were less closely related to *P. endodontalis* (66.4% and 66.2% ANI). To ascertain the phylogenetic placement of Candidatus taxa, a tree was produced of species representatives (Fig. 2a, Supplementary Fig. S3), revealing that the genomes identified here place near known human pathogens with standing in nomenclature: *P. uenonis*^46^ and *P. endodontalis*^47^. Expanding these investigations to other genera also revealed that two unknown genomes from Cluster GCA-2749205 within the *Treponemaceae* family also originated from a shared species.

We believe that the use of alpha-numeric codes for uncultivated taxa prohibits their extensive characterisation^48^. Renaming these taxa has been the subject of recent efforts^49^. As such, we have designated names to these *Candidatus* species (Supplementary Data 5). These include: *Ca*. Treponema oralis, *Ca*. Campylobacter oralis, *Ca*. Treponema bacchus, *Ca*. Porphyromonas dismagna, *Ca*. Porphyromonas severus, *Ca*. Treponema simulare, *Ca*. Enteromonas oralis, *Ca*. Capnocytophaga oralis, *Ca*. Porphyromonas leptis. *Ca*. Periomorbus oralis, *Ca*. Periomorbus coagulans, *Ca*. Nanosyncoccus oralis, *Ca*. Nanosyncoccus simulare, *Ca*. Nanosyncoccus coagulans, *Ca*. Nanosyncoccus arginin, *Ca*. Nanosyncoccus prostatocharitus.

As a proof of principle, we devised a method to develop and test species-specific PCR primers for one of the *Candidatus* species: ‘*Candidatus Porphyromonas leptis* sp. nov’ (EGAF00001796765.16) (methods). Briefly, the method includes identifying coding sequences for previously unidentified proteins, before taking primers forward for melt-curve analysis and specificity testing. PCR amplicon products of the predicted size were confirmed by agarose gel electrophoresis and Sanger sequencing confirmed the expected sequence for ‘*Candidatus Porphyromonas leptis* sp. nov’ (Supplementary Fig. S4).

67 phenotypic traits of the 15 putative unknown genomes were interrogated with Traitar3^50^. The top three most commonly observed phenotypic traits amongst genomes investigated include growth on ordinary blood agar (38/43 genomes investigated), Gram-negative (39/43 genomes) and bile susceptibility (36/43 genomes). 9/15 unknown genomes were predicted to grow under anaerobic conditions and 13 were predicted Gram-negative. Only three genomes were predicted Gram-positive. These were known species from the genera *Stomatobaculum* and *Mobiluncus and Rothia*. Within *Porphyromonas*, each of the putative unknown species were ubiquitously positive for the phenotypes: alkaline phosphatase, bile susceptibility, growth on blood agar, bacillus or coccobacillus, Gram-negative, anaerobic, casein hydrolysis, gelatin hydrolysis, traits expected of *Porphyromonas* species^46,51–53^. A table of all traits can be found in Supplementary Data 4. MAGs from shared genera share similar or identical functional trait prediction (Supplementary Fig. S5). Protologues for these taxa, supported by functional trait prediction as well as ENA accession IDs are provided in Supplementary Data 5.

### Predicted phenotypic traits associated with *Porphyromonas*

We sought to identify phenotypic traits associated with the ABBS genera, perhaps identifying candidates with a common contribution to the carcinogenic process. The Boruta feature selection algorithm was used to identify the phenotypic traits that hold predictive value for *Porphyromonas* genomes (including unknown *Candidatus* taxa) compared to other genomes. This identified 32 phenotypic traits that could be used to distinguish *Porphyromonas* (Supplementary Fig. S5). Fishers’ exact tests (Benjamini-Hochberg corrected) were used on these traits to identify nine phenotypic traits that are characteristics of *Porphyromonas* (adjusted *P-*value < 0.05): gelatin hydrolysis, arginine hydrolase, alkaline phosphatase, casein hydrolysis, Gram-negative, bile susceptibility, anaerobic growth, Bacillus or coccobacillus, growth on ordinary blood agar and indole production.

To identify other bacteria containing these features, we carried out Non-Metric Multidimensional Scaling (NMDS) on predicted phenotypic traits (Supplementary Fig. S5) and extracted genomes near *Porphyromonas* with similar traits (methods). This revealed the following genera with phenotypic traits similar to *Porphyromonas*: *Anaeroglobus*, *Prevotella*, *Tannerella*, cluster F0058 within the *Paludibacteraceae* family, *Bacteroides*, *Alloprevotella*, *Treponema*, cluster JABCPE02 within the *Weeksellaceae* family, *Dialister*, *Cryptobacteroides*, *Centipeda* and *Capnocytophaga*.

### Assembly identifies fewer anaerobic bacteria than short read classification

As well as the initial screening with Kraken, we employed a taxonomic profiler (mOTUs^54,55^ that has been previously benchmarked on tumour WGS data^23^) to search for evidence of genera in the ABBS. 170/247 (68.8%) of samples have an overlap in detecting at least one ABBS genera between Kraken and mOTUs2. As expected, Kraken identifies the most^23^, followed by metagenomic assembly (considering any quality of MAG). 97/427 (39.3%) of samples had at least one ABBS taxa that was recovered by metagenomic assembly.

### Shell orthogroups identifies genomic features of pathogenic *Porphyromonas* species

To characterise *Porphyromonas* species, Orthofinder was used to identify orthogroups shared between *N* = 66 *Porphyromonas* genomes including GTDB species and genomes identified in this study. *N* = 9314 orthogroups were identified. 381 were classified as core orthogroups (shared between all genomes), 341 soft-core orthogroups (95–99% of genomes), 1557 shell orthogroups (15–95% of genomes) and 7035 cloud orthogroups (<15% of genomes). This highlights a large amount of genomic diversity within the *Porphyromonas* genus (Supplementary Fig. S7). To identify differences between Porphyromonas genomes, we investigated differences in the presence-absence of shell orthogroups (Fig. 2b). Interestingly, the genomes can be divided into two clusters, with the genomes identified in this study occurring in one cluster alongside many known human pathogens associated with dental and urogenital conditions (*i.e.* *P. gingivalis*^56^, *P. endodontalis*^57^, *P. uenonis*^46^ and *P. asaccharolytica*^46^). Fishers’ exact tests revealed 456 and 499 shell orthogroups significantly enriched (Benjamini-Hochberg adjusted *p* < 0.05) in cluster 1 and 2 respectively. Sequences from these significant orthogroups were investigated using EggNOG^58^. 190 and 241 KEGG pathways were identified from these orthogroups for cluster 1 and 2 respectively after 42 intersecting KEGG pathways between clusters were removed (supplementary Table 6).

Several predicted features were identified as only present in one of the clusters. Cluster one contains more species that are known human pathogens and are better adapted for inflammatory and anaerobic environments, demonstrating proteolytic pathways and pathways consistent with intracellular infection. Cluster two appears to contain more shell orthogroups for carbohydrate utilisation and possibly a comparatively greater aerotolerance (Table 3).

**Table 3:** Characterising differences between *Porphyromonas* species based on KEGG annotations of shell orthologues

Cluster 1—Proteolytic, anaerobic and intracellular	Characteristics
Internalin A	- Permits internalisation in epithelial cells^74^ - Associated with stress response to oxygen^75^ - Internalin-E-Cadherin in *P. gingivalis* drives proliferative signalling and chemoresistance in head and neck cancer^75,76^
lysine 2,3-aminomutase [EC:5.4.3.2] beta-lysine 5,6-aminomutase [EC:5.4.3.3] [EC:5.4.3.3] 3-aminobutyryl-CoA ammonia-lyase [EC:4.3.1.14] histidine ammonia-lyase [EC:4.3.1.3]	- Proteolytic activity previously reported in *P. gingivalis*^77–79^
butyryl-CoA dehydrogenase [EC:1.3.8.1] 3-hydroxybutyryl-CoA dehydrogenase [EC:1.1.1.157] electron transfer flavoprotein subunits	- Butyrate production^80^ is associated with several cancer types^80–82^
**Cluster 2—Carbohydrate metabolising and aerotolerance**	
glycogen phosphorylase/synthase [EC:2.4.1.1 2.4.1.11] branching enzyme [EC:2.4.1.18] 4-alpha-glucanotransferase [EC:2.4.1.25] beta-galactosidase [EC:3.2.1.23] beta-glucuronidase [EC:3.2.1.31] alpha-glucosidase [EC:3.2.1.20] alpha-L-fucosidase [EC:3.2.1.51] sialidase [EC:3.2.1.18] beta-mannosidase [EC:3.2.1.25] mannan endo-1,4-beta-mannosidase [EC:3.2.1.78] hyaluronoglucosaminidase [EC:3.2.1.35] hexosaminidase [EC:3.2.1.52] N-acetylneuraminate lyase [EC:4.1.3.3]	- Examples of increased carbohydrate metabolism found to be important in aetiology of periodontal disease^83^.
Superoxide dismutase [EC:1.15.1.1] Catalase [EC:1.11.1.6] Thiol-dependent peroxiredoxin NADPH:quinone reductase	Markers suggesting comparative aerotolerance to species in cluster 1^84^

## Discussion

Here, we have demonstrated the recovery of both known and putative unknown MAGs from whole genome sequencing (WGS) data from prostate cancer patients. We have shown that accurate representations of microbial genomes can be recovered from WGS data that was intended to sequence human DNA. Several putative unknown species have been discovered, providing substantive expansions to a variety of bacterial clades, some of which have previously been associated with aggressive prostate cancer.

A high degree of similarity was shown between our *P. endodontalis* MAG and the *P. endodontalis* representative genome. This suggests the possibility that other MAGs may be of good quality. However, ultimately isolation of the organism will be required to confirm this. All essential genes for related species *P. gingivalis* were identified with the exception of *dnaX*.

Although in this study we have produced several ‘high-quality’ MAGs, future isolation, long/hybrid read sequencing and laboratory-based validation is required to fully ascertain that the genomes are free from chimeric sequences and to robustly ascertain the genomic characteristics from isolates. This is particularly true with the single-sample assembly/binning approach used in this study which has been shown to impact assembly quality^59^. This is also an important task in establishing future species representatives based on accurate genomics. We have reported on the predicted phenotypic traits of our putative unknown genomes to assist future culture and isolation efforts. Additionally, we report a method to develop species specific primer sequences for unknown bacterial genomes and provide these primers for one *Candidatus* species as an example to facilitate future investigations on clinical samples. It is also of note that these approaches could be applied to other scenarios, including environmental metagenomics.

The inclusion of control samples is useful to mitigate the impact of contamination, however there still exists an overlap between common sequencing contaminants and biologically relevant clades such as *Escherichia*^26^ which can render investigations difficult. Here we have identified putative unknown species from clades that are frequently described as common sequencing contaminants such as *Sphingomonas*, which was assembled in eight separate tumour samples. We postulate that the expanding the clades of common sequencing contaminants would offer researchers with an improved ability to identify samples containing such contaminants. Future studies of bacteria identified here will be required in order to ascertain biological significance.

A large majority of the genomes with high completeness/low contamination were retrieved from saliva samples compared with tumour samples (Table 1). Although these species have been previously cultured from clinical samples and identified using short-read detection approaches^5^, the oral cavity holds a higher microbial biomass, providing an enhanced propensity to assemble microbial genomes in saliva samples compared with tumours. It has been shown that there is an association between prostate cancer and the gastro-intestinal tract, including both the gut^13^ and oral^14^ microbiome. A comprehensive characterisation of the unknown bacterial species associated with the gastrointestinal tract of prostate cancer patients is of paramount importance. This study expands on our understanding of the bacteria residing in the gastrointestinal tract in prostate cancer patients and permits more complete investigations into exactly what microbes are associated with clinically relevant variables in future.

Furthermore, there are many more MAGs identified in this study that were not deemed to have high-quality characteristics and the ABBS list may be non-exhaustive for genera associated with aggressive prostate cancer. Therefore, these investigations should be expanded to samples from prostate cancer patients that are negative for ABBS taxa and the presence of incomplete taxa re-investigated across all samples. This would aid in the refinement of clinically relevant biomarkers. Furthermore, we do not propose that metagenomic assembly is used clinically to recover microbial genomes as the human depletion may impact rates of genome recovery – the performance of this approach has not been assessed in this manuscript. Although we have not been able to directly assess performance, it appears that metagenomic assembly is a less sensitive approach than mOTUs or Kraken at identifying members of the ABBS and assembly performance can vary widely depending on the approach used and characteristics of the input dataset^60,61^. The advantages of metagenomic recovery presented in this study however, include uncovering diversity and permit gene level insights. Furthermore, we have shown that this can be done with no additional cost for collecting and sequencing additional samples.

Analysis of estimated phenotypic traits has revealed taxa with similar phenotypic traits to *Porphyromonas*: *Anaeroglobus*, *Prevotella*, *Tannerella*, cluster F0058 within the *Paludibacteraceae* family, *Bacteroides*, *Alloprevotella*, *Treponema*, cluster *JABCPE02* within the *Flavobacteriaceae* family, *Dialister*, *Cryptobacteroides*, *Centipeda* and *Capnocytophaga*. We propose that these taxa should be examined in downstream studies for functional overlap with other bacteria from the ABBS which may be clinically relevant to the development of aggressive prostate cancer.

Overall, our results expand the status of known species and permits more complete investigations into the microbes that exist in cancer patients. Some of the species originate from the anaerobic bacterial biomarker genera list (ABBS) Expanding on the constituent species within this list will assist in our ability to identify microbial genomic features associated with aggressive prostate cancer by providing more representative species to investigate. This is needed to investigate a potential causal relationship between bacteria and the pathogenesis of aggressive cancer. Future work should include investigations on ABBS negative samples and uncovering microbial diversity in prostate cancer will permit healthy vs disease comparisons. It is hoped that this will ultimately contribute to either improved prognostic models or potentially new therapeutic avenues for prostate cancer patients.

## Methods

### Pan-Prostate Cancer Group (PPCG) cohort details

The PPCG is an international consortium that has harmonised the processing and analysis of DNA WGS data as well as DNA methylation and RNA sequencing. The WGS cohort comprises 1172 prostate samples from 1001 prostate cancer donors and more complete details are provided in Jakobsdottir et al.^40^. To summarise, each tumour sample comprises fresh-frozen tissue and all samples included in these studies were sequenced using Illumina sequencing with a median coverage of 66X (interquartile range = 27X) for tumours and a median depth of 41X (interquartile range = 12X) for paired normal.

### Identifying microbial reads

BAM files were produced by the Wellcome Sanger Institute Mapping Pipeline (cgpmap), using the Burrows-Wheeler Aligner (BWA-MEM)^62,63^ tool to align WGS data to GRCh37 (patch release 13 with decoy 5 GRCh37.p13 with d5 - https://ftp.sanger.ac.uk/pub/cancer/dockstore/human/core_ref_GRCh37d5.tar.gz) to produce aligned BAM files. These BAM files from the PPCG were downloaded using the pyEGA3 download client (from datasets EGAD00001004182, EGAD00001003835, EGAD00001004170, EGAD00001005997, EGAD00001004477, EGAD00001006742). Unmapped reads were extracted and merged with samtools (version 1.11). BAM files were converted to FASTQ using SamToFastq (Picard version 2.24.1). Unmapped reads were quality trimmed with Trimmomatic (version 0.39) with command “ILLUMINACLIP:bin/adapters_pe.fa:2:30:10 SLIDINGWINDOW:4:20 MINLEN:36”. Single and paired end reads were retained and subject to computational human read depletion using BBDuK (parameters *k* = 30, mcf = 0.5) and a database consisting of human reference GRCh38 (without EBV decoy sequences) and all CDS sequences for all genes in COSMIC (https://cancer.sanger.ac.uk/cosmic/download). The remaining sequencing reads (from both paired-end reads and single-ended reads that became unpaired during trimming) were classified with Kraken2 (confidence = 0.2). The Kraken2 database used was shared by Ben Langmead and downloaded using: “wget -c https://genome-idx.s3.amazonaws.com/kraken/k2_pluspfp_20210127.tar.gz” (md5sum= #b63fa1a51600ce3f0461578ce24301cc). Classified sequencing reads were statistically redistributed to genus level using Bracken using either “-r 100” or “-r 150” depending on the read length protocol used for sequencing. Redistributed classification files were merged using a custom R script to form a genus level community matrix. Samples were considered positive for ABBS if they had a minimum of 10 sequencing reads assigned to any genera within the biomarker set (*Anaerococcus*, *Porphyromonas*, *Ezakialla*, *Fenollaria*, *Fusobacterium*, *Porphyromonas*). A taxonomic profiler, mOTUS^55^ (version 3.1.0) was employed on unmapped reads (with sensitive parameters: -t 10 -g 1 -v 3).

### Metagenomic assembly

Data from samples where there was any level of evidence for an ABBS genera were taken forward for metagenomic assembly. 247 samples were subject to single sample assembly with MetaSPAdes^37^ (version 3.14) using standard parameters. This produced 10.2 million contigs. Coverage and the length of contigs were obtained from the headers provided in MetaSPAdes output files.

The resulting contigs in each sample were binned using Metabat (version 2.15-6)^64^ with a minimum contig size of 2000 and all other settings as default. Contamination and Completeness were assessed with CheckM^30^ (version 1.1.2) and CheckM2 (version 1.0.2)^29^. We applied GUNC^31^ to each of the MAGs with high completeness/low contamination as an orthogonal check for chimerism. All the putative unknown species passed suggesting minimal evidence for chimerism. Thirteen of these genomes from identified reference species failed quality checks with GUNC (version 1.0.5) with default parameters. Genomes were annotated with Bakta (version 1.9.4, default parameters)^65^. The number of tRNA and rRNA genes were approximated from nucleotide FASTA files using grep for ‘tRNA’ and ‘23S rRNA\|16S rRNA\|5S rRNA’. While this method provides an approximate count it does not determine the existence of complete rRNA operons.

### Taxonomic classification, phylogenetics and trait prediction

The classification workflow of GTDB-Tk^39^ was applied on all MAGs with high completeness and low contamination estimates (version 2.3.0, release 214). Microbial traits were inferred by Traitar3^50^ (version 0.1.0). NMDS was carried out using metaMDS (vegan R package, version 2.6.2) with parameters *k* = 2, distance = ‘jaccard’, try = 1000, trymax = 10,000. Analysing presence-absence of traits using dimensionality reduction demonstrated overlaps in microbial traits were observed for isolates from the same genus, providing another level of evidence that the recovered genomes are not overtly chimeric. (Supplementary Data 4, Supplementary Fig. S5).

To identify phenotypic traits characteristic that might be predictive of the *Porphyromonas* genomes that we recovered, a machine learning, feature selection algorithm (Boruta^66^) was employed on Traitar3 results, to predict whether a genome was from the *Porphyromonas* genus or not, using any genome with estimated medium or high-quality characteristics (CheckM) (random seed = 1, maxRuns = 10,000). Associated phenotypic traits were further identified using *Porphyromonas* and non-*Porphyromonas* genomes using Fishers’ exact tests with Benjamini-Hochberg multiple testing correction. To identify genomes most similar to *Porhyromonas* in terms of their phenotypic traits, the Traitar3 results were subject to NMDS (metaMDS, *k* = 2, trymax = 100), using the Jaccard distance (vegdist). Genomes close to a *Porphyromonas* genome were extracted within 0.2 units on the NMDS plot to identify genomes with similar phenotypic traits.

Orthologous Average Nucleotide Identity (OrthoANI^67^) was used to investigate phylogenetic relationships between members of the same genera. Focusing on orthologues makes OrthoANI particularly suited to investigating phylogenetic relationships between MAGs, which may have gaps, undetected chimerism and horizontal gene transfer.

Putative unknown genomes were annotated with Prokka^68^ (version 1.14.6). Mashtree^69^ (version 0.33) was ran on the resulting annotated nucleotide FASTA and GFF files. Orthologous average nucleotide identity was carried out with OAT (version 1.40) using the guided user interface to produce plots and the Java command line interface to permit comparisons of more than 10 genomes^67^.

PhyloPhlAn (version 3.1.68)^70^ was ran on all high quality metagenome assembled genomes (Fig. 1, parameters: --accurate –diversity high) or on Porphyromonas genomes (Fig. 2, parameters: --diversity medium). Tree files were produced with IQ-TREE3^71^ (version 3.1.1, parameters: -m MFP, -B 1000 -T AUTO). Plots were produced using the ggtree and ggtreeExtra R packages.

Orthofinder was used to identify orthologues within *Porphyromonas* species (default settings). *N* = 9314 orthogroups were found, 381 were classified as core orthogroups (shared between all genomes), 341 soft-core orthogroups (95–99% of genomes), 1557 shell orthogroups (15–95% of genomes) and 7035 cloud orthogroups (<15% of genomes). Hierarchical clustering was performed based on shell-gene presence-absence using the hclust and cutree functions. Orthogroups between the two clusters were investigated using Benjamini-Hochberg adjusted Fishers’ tests revealing 456 and 459 orthogroups significantly enriched in clusters 1 and 2, respectively. Orthogroup sequences differentially present between clusters were analysed using EggNOG^58^.

190 and 241 KEGG pathways were identified from these orthogroups for clusters 1 and 2, respectively after 42 intersecting KEGG pathways between clusters were removed (Supplementary Table 6). Resulting KEGG pathways were interpreted using the keggGet function (KEGGREST version 1.5).

### Primer design

A method was devised to develop specific PCR primers for the unknown species. Firstly, we annotated bacterial genomes using Bakta^65^. The resulting nucleotide FASTA file for all predicted transcripts were searched using BLAST^72^ against the refseq nucleotide database^73^. All transcripts that failed to obtain any hits were extracted (median of 715 per genome). These were further filtered with BBDuK to remove short sequences (minlength = 400), those with low complexity (entropy = 0.9) and those with constituent *k*-mers also present in the human genome (GRCh38). Three genes were selected at random for each genome and ten primer pairs were calculated using primerblast with an expected product size between 150 and 180 bp. Each resulting product was also checked for BLAST hits. Following this, a total of seven pairs of primers were taken forward to assess the optimal annealing temperature and specificity was checked against closely related bacterial isolates and mixed human samples. DNA from the original samples was used as a positive control (amplified using Qiagen Repli G protocol as per manufacturer instructions). Cycling conditions to check annealing temperatures in the range 51–63 °C were tested in triplicate for each primer pair, with a 2 °C increment in each test. Following PCR amplification, the amplicons were run on an 1.8% agarose gel stained with SYBR safe for 50’ and visualised under fluorescent light. Based on the intensity of the amplicon bands, 57 °C was set as the annealing temperature for primer pairs for ‘*Candidatus Porphyromonas leptis* sp. nov*’*. Agarose gel confirmations for positive control (amplified original DNA extract for EGAF00001796765 can be found in Supplementary Fig. S4).

This approach was successful for ‘*Candidatus Porphyromonas leptis* sp. nov’ (EGAF00001796765.16). The successful primer pair sequence and its 153 bp expected product sequence is as follows:

- Forward: GGGCAACAGCTTTCGTATCG, reverse: CGTATCCCTCCGCTCATAGG
- Expected product: GGGCAACAGCTTTCGTATCGCTTCTTGGTAACGGAAGCGCACCGTCCCTACTATTTCGACCTTCTTATTCGGCATAATAACAATGTAGAGTATTGTGGTTTTGATGTGCTGGCCACGCTTAGCGATAGCGCATCCTATGAGCGGAGGGATACG

## Supplementary information

Supplementary Information

Supplementary_Data

## Data Availability

BAM files from the PPCG were accessed from EGA for datasets: EGAD00001004182, EGAD00001003835, EGAD00001004170, EGAD00001005997, EGAD00001004477 and EGAD00001006742. Putative novel genomes can be found on the European Nucleotide Archive (ENA) with the accession PRJEB72026. Conversions between EGA identifiers and ENA identifiers can be found in Supplementary Data S5.

## Code availability

Processing scripts associated with this analysis can be found at on Zenodo with the DOI: 10.5281/zenodo.13819786

## References

[1] Bray, F. et al. Global cancer statistics 2022: GLOBOCAN estimates of incidence and mortality worldwide for 36 cancers in 185 countries. *CA Cancer J. Clin.* **74**, 229–263 (2024). 10.3322/caac.21834 38572751

[2] Hanahan, D. Hallmarks of cancer: new dimensions. *Cancer Discov.* **12**, 31–46 (2022). 10.1158/2159-8290.CD-21-1059 35022204

[3] Shrestha, E. et al. Profiling the urinary microbiome in men with positive versus negative biopsies for prostate cancer. *J. Urol.* **199**, 161–171 (2018). 10.1016/j.juro.2017.08.001 PMC5937117 28797714

[4] Salachan, P. V. et al. Microbiota of the prostate tumor environment investigated by whole-transcriptome profiling. *Genome Med.* **14**, 9 (2022). 10.1186/s13073-022-01011-3 PMC8787950 35078527

[5] Hurst, R. et al. Microbiomes of urine and the prostate are linked to human prostate cancer risk groups. *Eur. Urol. Oncol.* 10.1016/j.euo.2022.03.006 (2022). 35450835

[6] Shrestha, E. et al. Oncogenic gene fusions in nonneoplastic precursors as evidence that bacterial infection can initiate prostate cancer. *Proc. Natl. Acad. Sci. USA* **118**, e2018976118 (2021). 10.1073/pnas.2018976118 PMC8364155 34341114

[7] Benafif, S. et al. Germline sequencing in men with metastatic castration-resistant prostate cancer from the BARCODE2 study reveals a wide range of pathogenic variants in DNA repair genes. *BJC Rep.* **2**, 15 (2024). 10.1038/s44276-023-00024-8 PMC11507020 39516637

[8] Burns, D. et al. Rare germline variants are associated with rapid biochemical recurrence after radical prostate cancer treatment: a pan prostate cancer group study. *Eur. Urol.* 10.1016/j.eururo.2022.05.007 (2022). 35659150

[9] Hurst, R., Brewer, D. S., Gihawi, A., Wain, J. & Cooper, C. S. Cancer invasion and anaerobic bacteria: new insights into mechanisms. *J. Med. Microbiol.* **73**, 001817 (2024). 10.1099/jmm.0.001817 PMC10995961 38535967

[10] Zeng, X.-T. et al. Periodontal disease and incident lung cancer risk: a meta-analysis of cohort studies. *J. Periodontol.* **87**, 1158–1164 (2016). 10.1902/jop.2016.150597 27294431

[11] Maisonneuve, P., Amar, S. & Lowenfels, A. B. Periodontal disease, edentulism, and pancreatic cancer: a meta-analysis. *Ann. Oncol.* **28**, 985–995 (2017). 10.1093/annonc/mdx019 28453689

[12] Zeng, X. T. et al. Periodontal disease and risk of head and neck cancer: a meta-analysis of observational studies. *PLoS ONE* **8**, e79017 (2013). 10.1371/journal.pone.0079017 PMC3806857 24194957

[13] Pernigoni, N. et al. Commensal bacteria promote endocrine resistance in prostate cancer through androgen biosynthesis. *Science* **374**, 216–224 (2021). 10.1126/science.abf8403 34618582

[14] Guo, Z. et al. Periodontal disease and the risk of prostate cancer: a meta-analysis of cohort studies. *Int. Braz. J. Urol.* **47**, 1120–1130 (2021). 10.1590/S1677-5538.IBJU.2020.0333 PMC8486441 33650836

[15] Mysak, J. et al. Porphyromonas gingivalis: major periodontopathic pathogen overview. *J. Immunol. Res.* **2014**, 476068 (2014). 10.1155/2014/476068 PMC3984870 24741603

[16] Liss, M. A. et al. Metabolic biosynthesis pathways identified from fecal microbiome associated with prostate cancer. *Eur. Urol.* **74**, 575–582 (2018). 10.1016/j.eururo.2018.06.033 PMC6716160 30007819

[17] Sha, S., Ni, L., Stefil, M., Dixon, M. & Mouraviev, V. The human gastrointestinal microbiota and prostate cancer development and treatment. *Investig. Clin. Urol.* **61**, S43–S50 (2020). 10.4111/icu.2020.61.S1.S43 PMC7004837 32055753

[18] Alanee, S. et al. A prospective study to examine the association of the urinary and fecal microbiota with prostate cancer diagnosis after transrectal biopsy of the prostate using 16sRNA gene analysis. *Prostate* **79**, 81–87 (2019). 10.1002/pros.23713 30117171

[19] Golombos, D. M. et al. The role of gut microbiome in the pathogenesis of prostate cancer: a prospective, pilot study. *Urology* **111**, 122–128 (2018). 10.1016/j.urology.2017.08.039 28888753

[20] International Cancer Genome Consortium. International network of cancer genome projects. *Nature* **464**, 993–998 (2010). 10.1038/nature08987 PMC2902243 20393554

[21] The Cancer Genome Atlas Research Network The cancer genome atlas pan-cancer analysis project. *Nat. Genet* **45**, 1113–1120 (2013). 10.1038/ng.2764 PMC3919969 24071849

[22] Borchmann, S. An atlas of the tissue and blood metagenome in cancer reveals novel links between bacteria, viruses and cancer. *Microbiome* **9**, 94 (2021). 10.1186/s40168-021-01039-4 PMC8063312 33888160

[23] Gihawi, A. et al. SEPATH: benchmarking the search for pathogens in human tissue whole genome sequence data leads to template pipelines. *Genome Biol.* **20**, 208 (2019). 10.1186/s13059-019-1819-8 PMC6805339 31639030

[24] Gihawi, A. et al. Major data analysis errors invalidate cancer microbiome findings. *MBio* e0160723 (2023) 10.1128/mbio.01607-23. PMC10653788 37811944

[25] Gihawi, A., Cooper, C. S. & Brewer, D. S. Caution regarding the specificities of pan-cancer microbial structure. *Microb. Genom.* **9**, 001088 (2023). 10.1099/mgen.0.001088 PMC10483429 37555750

[26] Salter, S. J. et al. Reagent and laboratory contamination can critically impact sequence-based microbiome analyses. *BMC Biol.* **12**, 87 (2014). 10.1186/s12915-014-0087-z PMC4228153 25387460

[27] de Goffau, M. C. et al. Recognizing the reagent microbiome. *Nat. Microbiol.* **3**, 851–853 (2018). 10.1038/s41564-018-0202-y 30046175

[28] Knight, R. et al. Best practices for analysing microbiomes. *Nat. Rev. Microbiol.* **16**, 410–422 (2018). 10.1038/s41579-018-0029-9 29795328

[29] Chklovski, A., Parks, D. H., Woodcroft, B. J. & Tyson, G. W. CheckM2: a rapid, scalable and accurate tool for assessing microbial genome quality using machine learning. *Nat. Methods* **20**, 1203–1212 (2023). 10.1038/s41592-023-01940-w 37500759

[30] Parks, D. H., Imelfort, M., Skennerton, C. T., Hugenholtz, P. & Tyson, G. W. CheckM: assessing the quality of microbial genomes recovered from isolates, single cells, and metagenomes. *Genome Res* **25**, 1043–1055 (2015). 10.1101/gr.186072.114 PMC4484387 25977477

[31] Orakov, A. et al. GUNC: detection of chimerism and contamination in prokaryotic genomes. *Genome Biol.* **22**, 178 (2021). 10.1186/s13059-021-02393-0 PMC8201837 34120611

[32] Stewart, R. D. et al. Compendium of 4,941 rumen metagenome-assembled genomes for rumen microbiome biology and enzyme discovery. *Nat. Biotechnol.* **37**, 953–961 (2019). 10.1038/s41587-019-0202-3 PMC6785717 31375809

[33] Zeng, S. et al. A compendium of 32,277 metagenome-assembled genomes and over 80 million genes from the early-life human gut microbiome. *Nat. Commun.* **13**, 5139 (2022). 10.1038/s41467-022-32805-z PMC9437082 36050292

[34] Parks, D. H. et al. Recovery of nearly 8,000 metagenome-assembled genomes substantially expands the tree of life. *Nat. Microbiol.* **2**, 1533–1542 (2017). 10.1038/s41564-017-0012-7 28894102

[35] Pasolli, E. et al. Extensive unexplored human microbiome diversity revealed by over 150,000 genomes from metagenomes spanning age, geography, and lifestyle. *Cell* **176**, 649–662.e20 (2019). 10.1016/j.cell.2019.01.001 PMC6349461 30661755

[36] Carlino, N. et al. Unexplored microbial diversity from 2,500 food metagenomes and links with the human microbiome. *Cell* **187**, 5775–5795.e15 (2024). 10.1016/j.cell.2024.07.039 39214080

[37] Nurk, S., Meleshko, D., Korobeynikov, A. & Pevzner, P. A. metaSPAdes: a new versatile metagenomic assembler. *Genome Res* **27**, 824–834 (2017). 10.1101/gr.213959.116 PMC5411777 28298430

[38] Bowers, R. M. et al. Minimum information about a single amplified genome (MISAG) and a metagenome-assembled genome (MIMAG) of bacteria and archaea. *Nat. Biotechnol.* **35**, 725–731 (2017). 10.1038/nbt.3893 PMC6436528 28787424

[39] Chaumeil, P. A., Mussig, A. J., Hugenholtz, P. & Parks, D. H. GTDB-Tk: a toolkit to classify genomes with the Genome Taxonomy Database. *Bioinformatics*10.1093/bioinformatics/btz848 (2019). PMC7703759 31730192

[40] Jakobsdottir, G. M. et al. The Pan Prostate Cancer Group Dataset - Genomic, Transcriptomic and Methylation data for Clinical Discovery. *Sci. Data*10.1038/s41597-026-08181-4.

[41] Gihawi, A. et al. The landscape of microbial associations in human cancer. *Sci. Transl. Med.* **17**, eads6166 (2025). 10.1126/scitranslmed.ads6166 40901921

[42] Guilloux, C. A., Lamoureux, C., Beauruelle, C. & Hery-Arnaud, G. Porphyromonas: a neglected potential key genus in human microbiomes. *Anaerobe* **68**, 102230 (2021). 10.1016/j.anaerobe.2020.102230 32615270

[43] Wang, S.-Y. et al. P. gingivalis in oral-prostate axis exacerbates benign prostatic hyperplasia via IL-6/IL-6R pathway. *Mil. Med. Res.* **11**, 30 (2024). 10.1186/s40779-024-00533-8 PMC11103868 38764065

[44] Darling, A. E., Mau, B. & Perna, N. T. progressiveMauve: multiple genome alignment with gene gain, loss and rearrangement. *PLoS One* **5**, e11147 (2010). 10.1371/journal.pone.0011147 PMC2892488 20593022

[45] Klein, B. A. et al. Identification of essential genes of the periodontal pathogen Porphyromonas gingivalis. *BMC Genom.* **13**, 578 (2012). 10.1186/1471-2164-13-578 PMC3547785 23114059

[46] Finegold, S. M. et al. Porphyromonas uenonis sp. nov., a Pathogen for Humans Distinct from P. asaccharolytica and P. endodontalis. *J. Clin. Microbiol.* **42**, 5298–5301 (2004). 10.1128/JCM.42.11.5298-5301.2004 PMC525138 15528728

[47] Lombardo Bedran, T. B. et al. Porphyromonas endodontalis in chronic periodontitis: a clinical and microbiological cross-sectional study. *J. Oral. Microbiol.* **4**, 10123 (2012). 10.3402/jom.v4i0.10123 PMC3253302 22232719

[48] Pallen, M. J. The status Candidatus for uncultured taxa of Bacteria and Archaea: SWOT analysis. *Int. J. Syst. Evol. Microbiol.* **71**, 005000 (2021). 10.1099/ijsem.0.005000 PMC8549269 34516368

[49] Pallen, M. J., Rodriguez-R, L. M. & Alikhan, N.-F. Naming the unnamed: over 65,000 Candidatus names for unnamed Archaea and Bacteria in the Genome Taxonomy Database. *Int. J. Syst. Evol. Microbiol.* **72**, 005482 (2022). 10.1099/ijsem.0.005482 36125864

[50] Weimann, A. et al. From genomes to phenotypes: traitar, the microbial trait analyzer. *mSystems* **1**, 10–1128 (2016). 10.1128/mSystems.00101-16 PMC5192078 28066816

[51] Whaley, D. N., Wiggs, L. S., Miller, P. H., Srivastava, P. U. & Miller, J. M. Use of presumpto plates to identify anaerobic bacteria. *J. Clin. Microbiol.* **33**, 1196–1202 (1995). 10.1128/jcm.33.5.1196-1202.1995 PMC228130 7615728

[52] Morsczeck, C. O., Drees, J. & Gosau, M. Lipopolysaccharide from Escherichia coli but not from Porphyromonas gingivalis induce pro-inflammatory cytokines and alkaline phosphatase in dental follicle cells. *Arch. Oral. Biol.* **57**, 1595–1601 (2012). 10.1016/j.archoralbio.2012.07.016 22959004

[53] Fournier, D. et al. Porphyromonas gulae sp. nov., an anaerobic, gram-negative coccobacillus from the gingival sulcus of various animal hosts. *Int. J. Syst. Evol. Microbiol* **51**, 1179–1189 (2001). 10.1099/00207713-51-3-1179 11411686

[54] Sunagawa, S. et al. Metagenomic species profiling using universal phylogenetic marker genes. *Nat. Methods* **10**, 1196–1199 (2013). 10.1038/nmeth.2693 24141494

[55] Milanese, A. et al. Microbial abundance, activity and population genomic profiling with mOTUs2. *Nat. Commun.* **10**, 1014 (2019). 10.1038/s41467-019-08844-4 PMC6399450 30833550

[56] Reyes, L. Porphyromonas gingivalis. *Trends Microbiol.* **29**, 376–377 (2021). 10.1016/j.tim.2021.01.010 33546976

[57] Full article: Porphyromonas endodontalis in chronic periodontitis: a clinical and microbiological cross-sectional study. https://www.tandfonline.com/doi/full/10.3402/jom.v4i0.10123. 10.3402/jom.v4i0.10123 PMC3253302 22232719

[58] Huerta-Cepas, J. et al. Fast genome-wide functional annotation through orthology assignment by eggNOG-Mapper. *Mol. Biol. Evol.* **34**, 2115–2122 (2017). 10.1093/molbev/msx148 PMC5850834 28460117

[59] Mattock, J. & Watson, M. A comparison of single-coverage and multi-coverage metagenomic binning reveals extensive hidden contamination. *Nat. Methods* **20**, 1170–1173 (2023). 10.1038/s41592-023-01934-8 37386187

[60] Mendes, C. I. et al. LMAS: evaluating metagenomic short de novo assembly methods through defined communities. *Gigascience* **12**, giac122 (2022). 10.1093/gigascience/giac122 PMC9795473 36576131

[61] Mende, D. R. et al. Assessment of metagenomic assembly using simulated next generation sequencing data. *PLoS One* **7**, e31386 (2012). 10.1371/journal.pone.0031386 PMC3285633 22384016

[62] Li, H. & Durbin, R. Fast and accurate short read alignment with Burrows-Wheeler transform. *Bioinformatics* **25**, 1754–1760 (2009). 10.1093/bioinformatics/btp324 PMC2705234 19451168

[63] Li, H. Aligning Sequence Reads, Clone Sequences and Assembly Contigs with BWA-MEM. arXiv 10.48550/arXiv.1303.3997 (2013).

[64] Kang, D. D., Froula, J., Egan, R. & Wang, Z. MetaBAT, an efficient tool for accurately reconstructing single genomes from complex microbial communities. *PeerJ* **3**, e1165 (2015). 10.7717/peerj.1165 PMC4556158 26336640

[65] Schwengers, O. et al. Bakta: rapid and standardized annotation of bacterial genomes via alignment-free sequence identification. *Microb. Genom.* **7**, 000685 (2021). 10.1099/mgen.0.000685 PMC8743544 34739369

[66] Boruta—A System for Feature Selection—Miron B. Kursa, Aleksander Jankowski, Witold R. Rudnicki, 2010. https://journals.sagepub.com/doi/abs/10.3233/FI-2010-288?casa_token=VVzPziPEHu0AAAAA:ttggg26NpnKE3xkmu81QKTPpAHxUKpBnHPRzNP7Kd2eEg4RNYpL_JhlRwodcQyy4JwbVxsrbqwG-.

[67] Lee, I., Ouk Kim, Y., Park, S. C. & Chun, J. OrthoANI: an improved algorithm and software for calculating average nucleotide identity. *Int. J. Syst. Evol. Microbiol.* **66**, 1100–1103 (2016). 10.1099/ijsem.0.000760 26585518

[68] Seemann, T. Prokka: rapid prokaryotic genome annotation. *Bioinformatics* **30**, 2068–2069 (2014). 10.1093/bioinformatics/btu153 24642063

[69] Katz, L. S. et al. Mashtree: a rapid comparison of whole genome sequence files. *J Open Source Softw.* **4**, 10–21105 (2019). 10.21105/joss.01762 PMC9380445 35978566

[70] Precise phylogenetic analysis of microbial isolates and genomes from metagenomes using PhyloPhlAn 3.0 | *Nat. Commun*. https://www.nature.com/articles/s41467-020-16366-7. 10.1038/s41467-020-16366-7 PMC7237447 32427907

[71] Wong, T. K. F. et al. IQ-TREE 3: phylogenomic inference software using complex evolutionary models. *Mol. Biol. Evol.* **117** 10.1093/molbev/msag117 (2026). PMC13191116 42085559

[72] Basic local alignment search tool—ScienceDirect. https://www.sciencedirect.com/science/article/pii/S0022283605803602.

[73] NCBI Reference Sequence (RefSeq): a curated non-redundant sequence database of genomes, transcripts and proteins | Nucleic Acids Research | Oxford Academic. https://academic.oup.com/nar/article/33/suppl_1/D501/2505241?guestAccessKey=. 10.1093/nar/gki025 PMC539979 15608248

[74] Mengaud, J., Ohayon, H., Gounon, P., Mège, R.-M. & Cossart, P. E-cadherin is the receptor for internalin, a surface protein required for entry of L. monocytogenes into epithelial cells. *Cell* **84**, 923–932 (1996). 10.1016/s0092-8674(00)81070-3 8601315

[75] Wang, H. et al. Bacterial internalins exploit E-cadherin to promote head and neck tumor metastasis and drug resistance. 10.64898/2026.04.20.719623 (2026).

[76] Schubert, W.-D. et al. Structure of internalin, a major invasion protein of listeria monocytogenes, in complex with its human receptor E-cadherin. *Cell* **111**, 825–836 (2002). 10.1016/s0092-8674(02)01136-4 12526809

[77] Brazeau, B. J., Gort, S. J., Jessen, H. J., Andrew, A. J. & Liao, H. H. Enzymatic activation of lysine 2,3-aminomutase from Porphyromonas gingivalis. *Appl. Environ. Microbiol.* **72**, 6402–6404 (2006). 10.1128/AEM.01143-06 PMC1563663 16957271

[78] Identification of a Novel Pyridoxal 5‘-Phosphate Binding Site in Adenosylcobalamin-Dependent Lysine 5,6-Aminomutase from Porphyromonas gingivalis | Biochemistry. https://pubs.acs.org/doi/full/10.1021/bi020255k. 10.1021/bi020255k 12093296

[79] Moradali, M. F., Ghods, S., Angelini, T. E. & Davey, M. E. Amino acids as wetting agents: surface translocation by Porphyromonas gingivalis. 10.1038/s41396-019-0360-9. PMC6775972 30783212

[80] Okumura, S. et al. Gut bacteria identified in colorectal cancer patients promote tumourigenesis via butyrate secretion. *Nat. Commun.* **12**, 5674 (2021). 10.1038/s41467-021-25965-x PMC8479117 34584098

[81] Yang, H. J. & Kim, J. H. Role of microbiome and its metabolite, short chain fatty acid in prostate cancer. *Investig. Clin. Urol.* **64**, 3–12 (2023). 10.4111/icu.20220370 PMC9834570 36629060

[82] Gui, Q. et al. The association between gut butyrate-producing bacteria and non-small-cell lung cancer. *J. Clin. Lab. Anal.* **34**, e23318 (2020). 10.1002/jcla.23318 PMC7439349 32227387

[83] Lietzan, A. D. et al. Microbial β-glucuronidases drive human periodontal disease etiology. *Sci. Adv.* **9**, eadg3390 (2023). 10.1126/sciadv.adg3390 PMC10162664 37146137

[84] Xie, H. & Zheng, C. OxyR activation in porphyromonas gingivalis in response to a hemin-limited environment. *Infect. Immun.* **80**, 3471–3480 (2012). 10.1128/IAI.00680-12 PMC3457554 22825453

