# Supplementary material for: Recovering anaerobic bacterial genomes in saliva, tumour and blood whole genome sequencing data from patients with prostate cancer
Source: NPJ Biofilms Microbiomes. 2026 Oct 20;12:205. doi: 10.1038/s41522-026-01140-2 (PMC13683340; doi:10.1038/s41522-026-01140-2)
Supplement: Supplementary file 1 [file 41522_2026_1140_MOESM1_ESM.docx]

**Supplementary Figures for “Recovering Anaerobic Bacterial Genomes in Saliva, Tumour and Blood Whole Genome Sequencing Data from Patients with Prostate Cancer” by Gihawi *et al*.**

**Contents**

**Supplementary Figure S 1** – Workflow overview.

**Supplementary Figure S 2** – Mauve alignment plot between *Porphyromonas endodontalis* reference and metagenome assembled genome with *P. endodontalis* reference genome.

**Supplementary Figure S 3** – Comparative Genomics for novel bacterial species with related species.

**Supplementary Figure S 4** – Species specific qPCR assay development for *Candidatus* Porphyromonas leptis sp. nov.

**Supplementary Figure S 5** – Analysis of phenotypic traits of *Candidatus* novel species and those related to Porphyromonas.

**Supplementary Figure S 6** – Upset plot demonstrating the identification of Anaerobic Bacterial Biomarker Set genera by Kraken, mOTUs and metagenomic assembly.

**Supplementary Data 1** - CheckM results (version1 and version 2), genome characteristics and MIMAG standards for Metagenome Assembled Genomes (MAGs) isolated in this study.

**Supplementary Data 2** - GTDB-Tk classification of N=216 MAGs with high completion and low contamination estimates.

**Supplementary Data 3** - Taxonomic assignments of N=249 MAGs with high-quality completion or contamination estimates (CheckM version 1 or 2) and their samples of origin.

**Supplementary Data 4** - Taxonomic assignments of N=249 MAGs with high-quality completion or contamination estimates (CheckM version 1 or 2) and their samples of origin.

**Supplementary Data 5** - Protologues for newly named species identified by analysis of metagenome-assembled genomes from donor samples within the Pan-Prostate Cancer Group. These are provided on the European Nucleotide Archive (ENA) under study ID: ERP156818.

Supplementary Figure S 1 – The workflow used in this study. Reads unmapped to the human genome for 274 samples were submitted to single sample assembly using MetaSPAdes. The resulting 10.2 million contigs were binned into putative genomes using MetaBAT. These were checked for completeness, contamination and chimerism using CheckM with MIMAG standards and GUNC. This provided 216 high quality genomes, 314 medium quality genomes and 825 low quality genomes. The high-quality genomes were classified using GTDB-Tk to reveal 15 novel genomes. Comparative genomics of these genomes revealed 9 distinct novel species. Campylobacter_B: cluster B within the Campylobacter genus, GCA-2749205: Cluster GCA-2749205 within the Treponemaceae family, Treponema_D: cluster D within the Treponema genus.

Supplementary Figure S 2 - Mauve alignment plot demonstrating Porphyromonas endodontalis metagenome assembled genome (EGAF00001796793.17) showed on the top track aligned against Porphyromonas endodontalis reference genome (GCF_000174815.1) on the bottom track. The two genomes were aligned and visualised using progressiveMauve. Gaps in alignments were exported using the Mauve software. 20 gaps were reported in both genomes including 4 regions with no alignments in the P. endodontalis reference genome. These regions were searched for in the P. endodontalis gff file, revealing 54/1677 annotated protein coding genes: cmk, dnaJ, miaA, murB, rho, lpdA, cas2, cas1b, topA, dnaX, cobU, cas1c, bioA, nrdG, lipA, lptC, traJ, traK, traM, traN, pepT, serC, ftsH, mutS, mnmH, mnmA, argS, rfbD, bioB, yajC, coaE, cbiB, rpmF, purD, prfA, pyrF, lepA, lpxB, surE, infA, ykgO, rpsM, rpsK, rpsD, rpoA, rplQ, typA, ribH, prmA, nifJ, mltG, nth, fabF, uvrA. Of these genes, 23 were predicted to be essential genes in Porphyromonas gingivalis[1]: cmk, dnaJ, murB, rho, topA, dnaX, serC, ftsH, mnmA, argS, yajC, coaE, prfA, lepA, lpxB, surE, infA, rpsM, rpsK, rpsD, rpoA, ribH, fabF. The Porphyromonas MAG was annotated (prokka v 1.13) Each of the essential genes above were identified except for dnaX. This suggests that the gaps in the P. endodontalis reference genome may be due to natural variation between strains.

Supplementary Figure S 3 - Phylogenetics for putative novel species compared with related species in the same genus. Values in the squares represent the orthologous average nucleotide identity (OAT) between two genomes. Figures produced with OAT[2]. Different genera are shown within each figure, including A) Porphyromonas, B) Campylobacter_B, C) Cluster GCA-2749205 within the Treponemaceae family, D) Capnocytophaga, E) Periomorbus and F) Nanosyncoccus (produced with OATCMD). Putative novel species are indicated with a ‘put_nov_’ prefix. Proposed names for novel species can be found in supplementary data 5.

**A)** *Porphyromonas*

**B)** *Campylobacter_B*

Five samples recovered a single species of *Campylobacter_B*. The heatmap on the left shows these genomes compared to each other and a few reference genomes (in terms of average nucleotide identity). The heatmap in the middle shows one of these putative novel MAGs compared to nine other *Campylobacter* reference genomes.

***C) GCA-2749205***

***D) Capnocytophaga***

E) Putative novel *Candidatus* *Periomorbus* species represent two distinct taxa. Orthologous average nucleotide identity between EGAF00001796785.22.fa and EGAF00001796805.5.fa is 93.94%.

F) *Nanosyncoccus*

Supplementary Figure S 4 – detection of species-specific qPCR assay amplicon product by agarose gel electrophoresis to identify putative novel bacterial species “Candidatus Porphyromonas leptis” (EGAF00001796729.56). DNA extracted from the saliva sample EGAF00001796729 was used in an amplification protocol using the Qiagen Repli G protocol as per manufacturer instructions. The amplified DNA was used as positive controls with triplicate replicates (A, B, C) for the development of a species-specific qPCR assay. The amplicons of the qPCR assay are shown at the expected product size (153bp), the sequence was confirmed as the expected sequence. Two non-template controls (D, E) were run for this primer pair, which produced no amplification.

A)	B)

C)

Supplementary Figure S 5 – **A)** Microbial traits of putative 18 genomes from putative Candidatus novel bacterial genomes assembled from PPCG prostate cancer data. Traits were predicted using Traitar3 and were subject to non-metric multidimensional scaling (vegan R package, metaMDS function, k=2, distance=’jaccard’, try=1000, trymax = 10000). The stress metric was 0.055 which indicates a good fit of the data in two dimensions. **B)** Phenotypic traits confirmed to hold value in predicting Porphyromonas compared to other genomes. **C)** NMDS plot of phenotypic traits for all microbial genomes with medium or high-quality characteristics. The following genera were identified within 0.2 units of a Porphyromonas genome: Anaeroglobus, Prevotella, Tannerella, cluster F0058 within the Paludibacteraceae family, Bacteroides, Alloprevotella, Treponema, cluster JABCPE02 within the Weeksellaceae family,, Dialister, Cryptobacteroides, Centipeda and Capnocytophaga.

Supplementary Figure S 6 - Upset plot demonstrating the identification of Anaerobic Bacterial Biomarker Set (ABBS) genera (Fenollaria, Ezakiella, Porphyromonas, Peptoniphilus, Fusobacterium, Anaerococcus) by three methods: Kraken, mOTUs and metagenomic assembly. Samples here were those initially deemed positive for at least one ABBS genera by Kraken (n=247).

Supplementary figure S7 – The distribution of *N*=9,314 orthogroups. 381 were classified as core orthogroups (shared between all genomes), 341 soft-core orthogroups (95-99% of genomes), 1,557 shell orthogroups (15-95% of genomes) and 7,035 cloud orthogroups (<15% of genomes).

A)

Supplementary figure S8 – Analysis of *Porphyromonas* species representatives. **A)** A phylogenetic tree of *Porphyromonas* species representatives (GTDB) and species isolated in this study rooted using *Tanerella forsythia*. The tree was generated by first checking completeness with CheckM2, aligning marker genes with PhyloPhlAn (version 3.1.68), generating a tree file with Iqtree3 (version 3.1.1) and plotting with the ggtree and ggtreeExtra R packages. **B)** Presence of shell orthogroups in *Porphyromonas* species representatives. Orthogroups and genomes were subject to hierarchical clustering and the genomes divided into two clusters (blue and red). Shell orthogroups were found using Orthofinder.

**Supplementary Data**

See the supplementary data file included with the article for the following datasets.

**Supplementary Data 1** – CheckM results (version1 and version 2), genome characteristics and MIMAG standards for Metagenome Assembled Genomes (MAGs) isolated in this study.

**Supplementary Data 2** – GTDB-Tk classification of N=216 MAGs with high completion and low contamination estimates.

**Supplementary Data 3** – Taxonomic assignments of N=249 MAGs with high-quality completion or contamination estimates (CheckM version 1 or 2) and their samples of origin.

**Supplementary Data 4** – Phenotypic trait prediction of Metagenome Assembled Genomes (MAGs) using Traitar3.

**Supplementary Data 5** - Protologues for newly named species identified by analysis of metagenome-assembled genomes from donor samples within the Pan-Prostate Cancer Group. These are provided on the European Nucleotide Archive (ENA) under study ID: ERP156818.

**Supplementary Data 6** - Kegg Pathways identified as differentially present between two clusters of Porphyromonas genomes

**References:**

[1] Klein BA, Tenorio EL, Lazinski DW, Camilli A, Duncan MJ, Hu LT. Identification of essential genes of the periodontal pathogen Porphyromonas gingivalis. BMC Genomics. 2012;13:578.

[2] Lee I, Ouk Kim Y, Park SC, Chun J. OrthoANI: An improved algorithm and software for calculating average nucleotide identity. Int J Syst Evol Microbiol. 2016;66:1100-3.
